# Supplementary material for: Hidden Markov modeling for maximum probability neuron reconstruction
Source: Commun Biol. 2022 Apr 25;5:388. doi: 10.1038/s42003-022-03320-0 (PMC9038756; doi:10.1038/s42003-022-03320-0)
Supplement: Supplementary file 2 — Supplementary Information [file 42003_2022_3320_MOESM2_ESM.pdf]

Supplement: Hidden Markov Modeling for Maximum Probability Neuron Reconstruction

Thomas L. Athey[1,2*], Daniel J. Tward[3,4], Ulrich Mueller[5], Joshua T. Vogelstein[1,2,6,7] and Michael I. Miller[1,2,6,7]

[1*]Department of Biomedical Engineering, Johns Hopkins University, 3400 N. Charles Street, Baltimore, 21218-2681, MD, USA.
[2]Institute of Computational Medicine, Johns Hopkins University, 3400 N. Charles Street, Baltimore, 21218-2681, MD, USA.
[3]Department of Computational Medicine, University of California at Los Angeles, BOX 951766, Room 5303 Life Sciences, Los Angeles, 90095-1766, CA, USA.
[4]Department of Neurology, University of California at Los Angeles, BOX 951766, Room 5303 Life Sciences, Los Angeles, 90095-1766, CA, USA.
[5]Department of Neuroscience, Johns Hopkins University, 725 N. Wolfe Street, Baltimore, 21205, MD, USA.
[6]Center for Imaging Science, Johns Hopkins University, 3400 N. Charles Street, Baltimore, 21218-2681, MD, USA.
[7]Kavli Neuroscience Discovery Institute, Johns Hopkins University, 3400 N. Charles Street, Baltimore, 21218-2681, MD, USA.

*Corresponding author(s). E-mail(s): tathey1@jhu.edu; Contributing authors: DTward@mednet.ucla.edu; umuelle3@jhmi.edu; jovo@jhu.edu; mim@cis.jhu.edu;

Supplementary Methods

Supplementary Method 1: Computing Endpoints and Tangents of Fragments

As explained in Section 4.2, each fragment is a subset of the image $F \subset D$. Each fragment is assumed to be associated with a segment of an underlying neuron curve, and we want to estimate the locations of the two endpoints of the fragment. First, we compute the length of the diagonal of the bounding box that contains the fragment. We divide this length by two to get R. Then, with each voxel y in the fragment, we associate a set of voxels N_y which is the intersection of the voxels in the fragment and the voxels within distance R from y. The voxel with the smallest set N_y (by cardinality) is chosen to be the first endpoint. The second endpoint is the voxel that has the smallest set N_y but is also farther than R away from the first endpoint.

Currently the tangents at the endpoints is just approximated by the difference of the endpoints i.e. $\tau^0 = \frac{x_0 - x_1}{||x_0 - x_1||}, \tau^1 = -\tau^1$. This method is based on the assumption that fragments are small enough that they are approximately straight. We also experimented with approximating the endpoint tangents by computing principal components of voxels near the endpoints, but found it to be less robust for downstream reconstruction.

Supplementary Method 2: Curvature Calculation for the Potential in the Markov Chain

The term $\kappa(s_{i-1}, s_i)$ is an approximation of the curvature of the path that connects s_{i-1} to s_i. For a curve with the tangent vector $T(s)$, curvature is defined as $\kappa(s) = \left|\left|\frac{dT}{ds}\right|\right|$. A finite difference approximation of curvature is then:

$$\begin{aligned}\kappa(s)^2 = \left|\left|\frac{dT}{ds}\right|\right|^2 &\approx ||T(s) - T(s-1)||^2 \\ &= ||T(s)||^2 + ||T(s-1)||^2 - 2T(s) \cdot T(s-1) \\ &= 2(1 - T(s) \cdot T(s-1))\end{aligned}$$

Thus,

$$\left|\left|\frac{dT}{ds}\right|\right|^2 \propto 1 - T(s+1) \cdot T(s) \tag{1}$$

We consider τ_i, τ_{i-1} and the normalized vector between the states $\tau_c := \frac{x^1_{i-1} - x^0_i}{|x^1_{i-1} - x^0_i|}$ as samples of $T(s)$, then use Eq. (1) to estimate the curvature induced by connecting state s_{i-1} to s_i:

$$\begin{aligned}(\kappa_1)^2 &= 1 - \tau_{i-1}^1 \cdot \tau_c \\ (\kappa_2)^2 &= 1 - \tau_c \cdot (-\tau_i^0) \\ \kappa(s_{i-1}, s_i)^2 &= \frac{(\kappa_1)^2 + (\kappa_2)^2}{2} \qquad \text{Arithmetic mean}\end{aligned}$$

Supplementary Method 3: Proofs

Recall the likelihood of a complete fragment under the foreground-background model (Eq. 3b):

$$\alpha_k(I_F) := \prod_{y \in F} \alpha_k(I_y) \ , k = 0, 1 \ .$$

Lemma 1:
For $n > 1$ we have the recursion probability

$$p(s_{1:n}, I_D) = \left(\frac{\alpha_1(I_{F_n})}{\alpha_0(I_{F_n})}\right)^{\delta_{D \setminus F_{1:n-1}}(F_n)} p(s_n | s_{n-1}) p(s_{1:n-1}, I_D) \tag{2a}$$

implying the factored probability:

$$p(s_{1:n}, I_D) = \prod_{i=2}^{n} \left(\frac{\alpha_1(I_{F_i})}{\alpha_0(I_{F_i})}\right)^{\delta_{D \setminus F_{1:i-1}}(F_i)} p(s_i | s_{i-1}) p(s_1, I_D) \tag{2b}$$

Proof Factor the event $I_D = (I_{F_{1:n}}, I_{D \setminus F_{1:n}})$, then

$$\begin{aligned}p(s_{1:n}, I_{F_{1:n}}, I_{D \setminus F_{1:n}}) &= p(I_{F_n} | s_{1:n}, I_{F_{1:n-1}}, I_{D \setminus F_{1:n}}) p(s_n | s_{1:n-1}, I_{D \setminus F_{1:n}}) p(s_{1:n-1}, I_{F_{1:n-1}}, I_{D \setminus [illegible]} \\ &= p(I_{F_n} | s_n)^{\delta_{D \setminus F_{1:n-1}}(F_n)} p(s_n | s_{n-1}) p(s_{1:n-1}, I_{F_{1:n-1}}, I_{D \setminus F_{1:n}}) \\ &= \alpha_1(I_{F_n})^{\delta_{D \setminus F_{1:n-1}}(F_n)} p(s_n | s_{n-1}) p(s_{1:n-1}, I_{F_{1:n-1}}, I_{D \setminus F_{1:n}})\end{aligned}$$

We rewrite the last term using the splitting property:

$$\begin{aligned}p(s_{1:n-1}, I_{F_{1:n-1}}, I_{D \setminus F_{1:n}}) &= p(I_{F_{1:n-1}} | s_{1:n-1}, I_{D \setminus F_{1:n}}) p(s_{1:n-1}, I_{D \setminus F_{1:n}}) \\ &= p(I_{F_{1:n-1}} | s_{1:n-1}) p(I_{D \setminus F_{1:n}} | s_{1:n-1}) p(s_{1:n-1}) \\ &= p(I_{F_{1:n-1}} | s_{1:n-1}) \frac{p(I_{D \setminus F_{1:n-1}} | s_{1:n-1})}{p(I_{F_n} | s_{1:n-1})^{\delta_{D \setminus F_{1:n-1}}(F_n)}} p(s_{1:n-1}) \\ &= \frac{1}{\alpha_0(I_{F_n})^{\delta_{D \setminus F_{1:n-1}}(F_n)}} p(s_{1:n-1}, I_{F_{1:n-1}}, I_{D \setminus F_{1:n-1}})\end{aligned}$$

with the last substitution following from the background model. Substituting into 3 yields the probability written as a recursion 2a:

$$p(s_{1:n}, I_D) = \left(\frac{\alpha_1(I_{F_n})}{\alpha_0(I_{F_n})}\right)^{\delta_{D \setminus F_{1:n-1}}(F_n)} p(s_n | s_{n-1}) p(s_{1:n-1}, I_D)$$

□

Statement 1: Define the most probable solution $s_{1:n} \in \mathcal{S}^n$ by the joint probability
$\mathrm{argmax}_{s_{1:n} \in \mathcal{S}^n} p(s_{1:n}, I_{F_{1:n}})$. Then we have

$$\max_{s_{1:n} \in \mathcal{S}^n} p(s_{1:n}, I_{F_{1:n}}) = \max_{s_{1:n} \in \mathcal{S}^n} \prod\nolimits_{i=2}^{n} (\alpha_1(I_{F_i}))^{\delta_{D \setminus F_{1:i-1}}(F_i)} p(s_i|s_{i-1})\, p(s_1, I_{F_1}) \,. \tag{4}$$

Further, if $\alpha_1(I_y) \leq 1$ for all y, then the globally optimal solution to the fixed start and end point problem is a nonrepeating state sequence and can be obtained by computing the shortest path in a directed graph where the vertices are the states, and the edge weight from state s_{i-1} to s_i is given by:

$$e(s_{i-1}, s_i) = -\log \alpha_1(I_{F_i}) - \log p(s_i|s_{i-1}) \tag{5}$$

Proof First we will demonstrate the factorization in Eq. 4:

$$\begin{aligned} p(s_{1:n}, I_{F_{1:n}}) &= p(s_{1:n}, I_{F_{1:n-1}}, I_{F_n}) \\ &= p(I_{F_n}|s_{1:n}, I_{F_{1:n-1}}) p(s_{1:n}, I_{F_{1:n-1}}) \\ &= p(I_{F_n}|s_{1:n}, I_{F_{1:n-1}}) p(I_{F_{1:n-1}}|s_{1:n}) p(s_{1:n}) \\ &= p(I_{F_n}|s_{1:n}, I_{F_{1:n-1}}) p(I_{F_{1:n-1}}|s_{1:n-1}) p(s_n|s_{1:n-1}) p(s_{1:n-1}) \\ &= (\alpha_1(I_{F_n}))^{\delta_{D \setminus F_{1:n-1}}(F_n)} p(s_n|s_{n-1}) p(I_{F_{1:n-1}}|s_{1:n-1}) p(s_{1:n-1}) \,. \end{aligned}$$

Applying this to factor the conditional probability , $I_{F_{1:i}}$, $i = n-1, \ldots, 1$ gives the joint product. Next, we will show how this can be solved using shortest path algorithms if $\alpha_1(I_y) \leq 1$ for all y:

First, we want to show that the globally optimal sequence is nonrepeating. This is clear because if $\alpha_1(I_y) \leq 1$, then every term in the product in Equation 6 (Section 4.3) is bounded by 1. Thus, for any state sequence that repeats states, if we remove all elements between the two instances of the repeated states, then this new sequence will have at least as much probability $p(s_{1:n}, I_{F_{1:n}})$.

For nonrepeating state sequences, the probability $p(s_{1:n}, I_{F_{1:n}})$ of Equation 6 can be simplified:

$$p(s_{1:n}, I_{F_{1:n}}) = \prod\nolimits_{i=2}^{n} (\alpha_1(I_{F_i}))\, p(s_i|s_{i-1}) p(s_1, I_{F_1}) \,.$$

Taking the logarithm yields a sequentially additive cost function that can be solved with shortest path algorithm on a graph with edge weights given by Equation (5).

□

Supplementary Method 4: Viterbi Algorithm Counter-example

Here we present a simple counter example demonstrating that the indicator function in Equation 5 (Section 4.3) cannot be ignored, implying the globally

optimal solution cannot be obtained efficiently with the Viterbi algorithm. The details of the state likelihoods and transition probabilities are given in Supplementary Figure 1a.

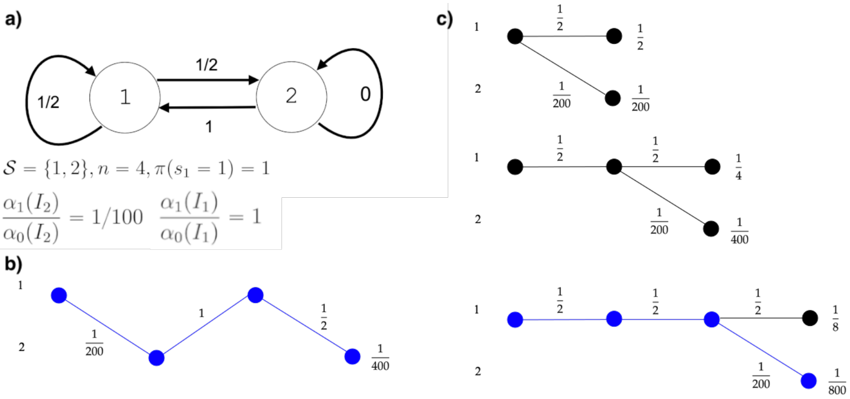

Supplementary Figure 1. Example showing that the Viterbi algorithm is invalid in our setting. a) Transition probabilities, and image likelihood ratios of a two state Markov model where, in our probabilistic model, the globally optimal state sequence from state 1 to state 2 with length 4 cannot be computed with the classic Viterbi algorithm. Panels **b)** and **c)** depict paths through the state space. The nodes represent the states and the line segments represent state transitions, as in Figure 8 of Forney [1]. **b)** True shortest path from state 1 to state 2 with length 4. **c)** Three iterations of the Viterbi algorithm, with the lines representing the paths that are stored at each iteration. In this example, the blue path at the bottom of panel **c)** is identified by naive application of the Viterbi algorithm, but it differs from the true shortest path in panel b). Each transition has a number above it indicating its contribution to the joint probability - $\left(\frac{\alpha_1(I_{F_i})}{\alpha_0(I_{F_i})}\right)^{\delta_{D \setminus F_{1:i-1}}(F_i)} p(s_i|s_{i-1})$ from Eqn. 5. The number at the end of the path is the product of all such terms - the joint probability of the given path.

Here, we remove the indicator function in 5 and apply the Viterbi algorithm in the usual way. After three iterations, the Viterbi algorithm stores $(1, 1, 1, 2)$ as the highest probability path of length 4 that ends in state 2 (Supplementary Figure 1c). The joint probability of this path is $1/800$. However, there is an alternative path that the algorithm missed, $(1, 2, 1, 2)$, which has higher joint probability $1/400$ (Supplementary Figure 1b). The algorithm did not identify the globally optimal sequence because it did not "see" that the cost of transitioning from state 1 to 2 would drop from $1/200$ to $1/2$ after visiting state 2 the first time.

Supplementary Figures

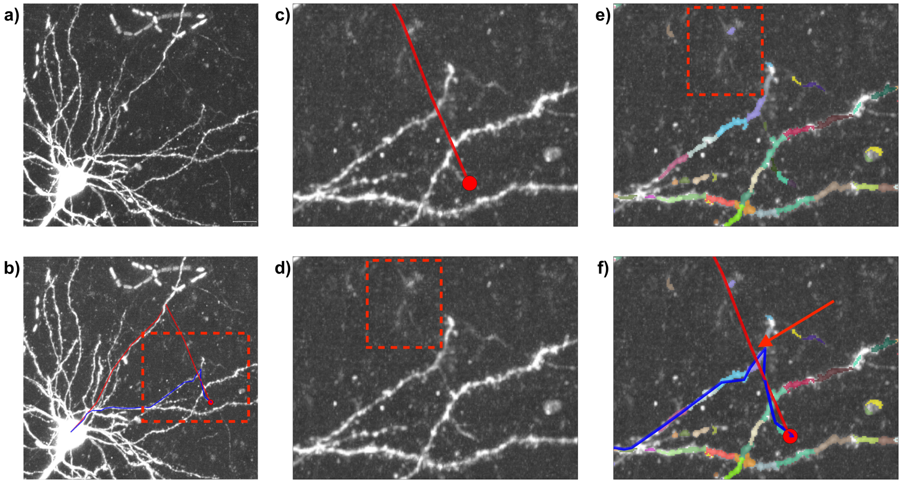

Supplementary Figure 2. The most common failure mode is an inadequate fragment space due to signal dropout. a) An image subvolumes from a MouseLight image. The scale bar represents 10 microns. **b)** The subvolume with the blue line depicting the most probable reconstruction and the red line depicting the manual reconstruction. The red dotted box shows the zoomed area that is depicted in panels **c-f)**. **c)** Close up view with the manual reconstruction in red. **d)** Close up view with red dotted box depicting a low (barely visible) image signal under the manual reconstruction. **e)** Close up view with the fragments in colors, showing that no fragments were generated in the area of weak signal. **f)** Close up view with both the manual and ViterBrain reconstruction, with the point of deviation shown with the red arrow.

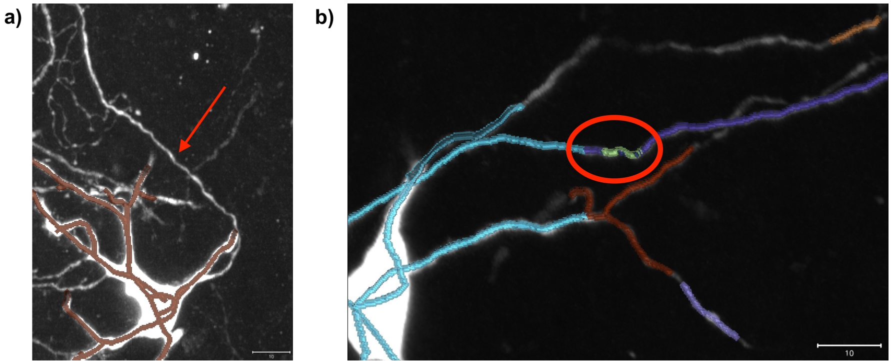

Supplementary Figure 3. GTree produced sensible reconstructions on our dataset, but often fell short of fully reconstructing the axon segment in question. We observed two common failure modes with this algorithm. First, the reconstruction would sometimes fail to extend far enough down the axon. **a)** Example where the GTree reconstruction (brown overlay) does not extend up the axon identified with the red arrow. **b)** Depiction of the other primary failure mode, when GTree splits a single axon into several components. Each colored overlay in **b)**, including green, blue, and purple represents a distinct reconstruction object produced by GTree. In other words, GTree severed the neuron (emphasized by the red circle) into several components and failed to fully reconstruct the axon. Both scale bars represent 10 microns.

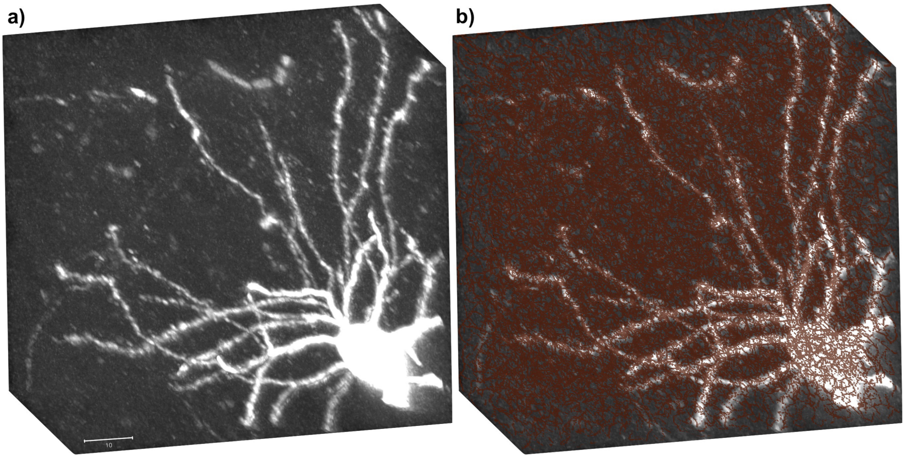

Supplementary Figure 4. The Snake tracing algorithm produced incoherent reconstructions on our dataset. a) One of the image subvolumes, with scale bar representing 10 microns. **b)** A reconstruction by Snake (brown overlay) which is a dense, convoluted set of tangled paths. This algorithm was applied to 10 different subvolumes and the results were similar each time. There were no options to change any parameters in the Vaa3D implementation of this algorithm, and the algorithm took over an hour to process any subvolume larger than 50 megabytes, so we terminated its use after only 10 subvolumes.

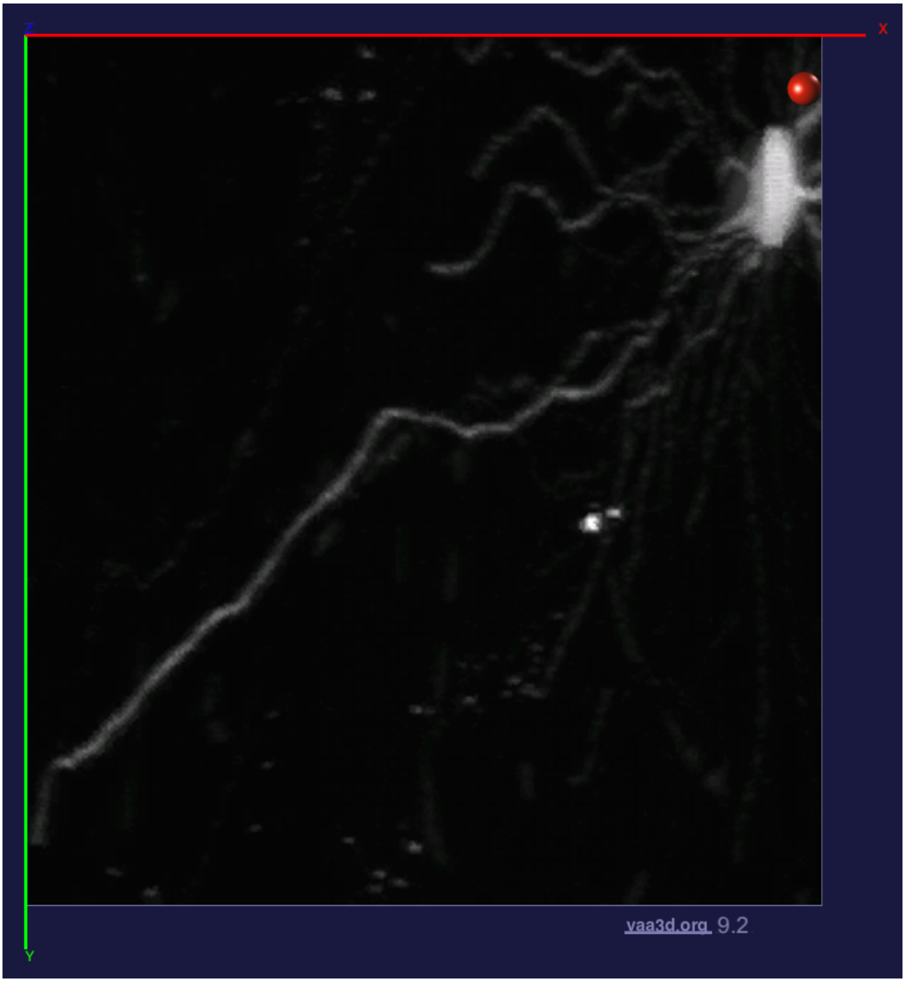

Supplementary Figure 5. The Advantra algorithm, as implemented in Vaa3D, did not produce coherent reconstructions on the test dataset. Even under different hyperparameter settings, the algorithm would often produce a reconstruction composed of only a single point, like the one shown here. Scale bar is in microns.

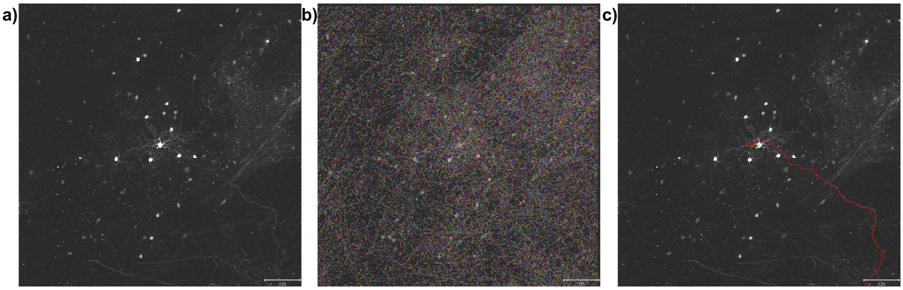

Supplementary Figure 6. We applied the ViterBrain pipeline to an image subvolume with dimensions $3332 \times 3332 \times 1000$ **voxels, which encompasses a cubic millimeter of tissue.** Pictured is a set of maximum intensity projections of a downsampled version of this subvolume. **a)** The image. **b)** The image with a color overlay depicting the neuron fragments. **c)** A trace in red that follows an axon. The scale bar represents 125 microns.

Supplementary References

[1] Forney, G.D.: The viterbi algorithm. Proceedings of the IEEE **61**(3), 268–278 (1973)
