# Supplementary material for: Hidden Markov modeling for maximum probability neuron reconstruction
Source: Commun Biol. 2022 Apr 25;5:388. doi: 10.1038/s42003-022-03320-0 (PMC9038756; doi:10.1038/s42003-022-03320-0)
Supplement: Supplementary file 4 — Reporting Summary [file 42003_2022_3320_MOESM4_ESM.pdf]

Corresponding author(s): Thomas Athey

Last updated by author(s): Feb 23, 2022

Reporting Summary

Nature Portfolio wishes to improve the reproducibility of the work that we publish. This form provides structure for consistency and transparency in reporting. For further information on Nature Portfolio policies, see our Editorial Policies and the Editorial Policy Checklist.

Statistics

For all statistical analyses, confirm that the following items are present in the figure legend, table legend, main text, or Methods section.

n/a	Confirmed	
☐	☒	The exact sample size (*n*) for each experimental group/condition, given as a discrete number and unit of measurement
☐	☒	A statement on whether measurements were taken from distinct samples or whether the same sample was measured repeatedly
☐	☒	The statistical test(s) used AND whether they are one- or two-sided<br>*Only common tests should be described solely by name; describe more complex techniques in the Methods section.*
☒	☐	A description of all covariates tested
☐	☒	A description of any assumptions or corrections, such as tests of normality and adjustment for multiple comparisons
☐	☒	A full description of the statistical parameters including central tendency (e.g. means) or other basic estimates (e.g. regression coefficient) AND variation (e.g. standard deviation) or associated estimates of uncertainty (e.g. confidence intervals)
☐	☒	For null hypothesis testing, the test statistic (e.g. *F*, *t*, *r*) with confidence intervals, effect sizes, degrees of freedom and *P* value noted<br>*Give P values as exact values whenever suitable.*
☒	☐	For Bayesian analysis, information on the choice of priors and Markov chain Monte Carlo settings
☒	☐	For hierarchical and complex designs, identification of the appropriate level for tests and full reporting of outcomes
☒	☐	Estimates of effect sizes (e.g. Cohen's *d*, Pearson's *r*), indicating how they were calculated

Our web collection on statistics for biologists contains articles on many of the points above.

Software and code

Policy information about availability of computer code

Data collection	The data used is from the Janelia Mouselight project, which already openly shares its neuron reconstructions. The specific data used in the paper is hosted and publicly accessible by our lab.
Data analysis	All software used is available in the most recent version of our open source Python package, brainlit, available at: http://brainlit.neurodata.io/

For manuscripts utilizing custom algorithms or software that are central to the research but not yet described in published literature, software must be made available to editors and reviewers. We strongly encourage code deposition in a community repository (e.g. GitHub). See the Nature Portfolio guidelines for submitting code & software for further information.

Data

Policy information about availability of data

All manuscripts must include a data availability statement. This statement should provide the following information, where applicable:

- Accession codes, unique identifiers, or web links for publicly available datasets
- A description of any restrictions on data availability
- For clinical datasets or third party data, please ensure that the statement adheres to our policy

The brain images come from a sample from the Janelia Mouselight project and is publicly accessible in our open neurodata bucket in AWS: s3://open-neurodata/brainlit/brain1

Field-specific reporting

Please select the one below that is the best fit for your research. If you are not sure, read the appropriate sections before making your selection.

☒ Life sciences ☐ Behavioural & social sciences ☐ Ecological, evolutionary & environmental sciences

For a reference copy of the document with all sections, see nature.com/documents/nr-reporting-summary-flat.pdf

Life sciences study design

All studies must disclose on these points even when the disclosure is negative.

Sample size	Our paper is primarily a methods paper that includes figures/data that qualitatively describe how the algorithm operates. However, we do perform a quantitative experiment where we measure the accuracy of 35 reconstructions. The answers below pertain to that experiment.
Data exclusions	As explained in the caption of Figure 7 and in the Suppement, one of the algorithms (Snake) was only applied to part of the dataset, since it always produced incoherent results, and had excessively long runtimes.
Replication	We present results on a single dataset of 35 image subvolumes.
Randomization	The neurons in the dataset correspond to the first 35 neurons in a collection of reconstructions from one whole-brain image. All algorithms are applied to the same subvolumes.
Blinding	The results were not blinded - the same experimenter (TA) applied all the algorithms to all the subvolumes.

Reporting for specific materials, systems and methods

We require information from authors about some types of materials, experimental systems and methods used in many studies. Here, indicate whether each material, system or method listed is relevant to your study. If you are not sure if a list item applies to your research, read the appropriate section before selecting a response.

Materials & experimental systems

n/a	Involved in the study	
☒	☐	Antibodies
☒	☐	Eukaryotic cell lines
☒	☐	Palaeontology and archaeology
☐	☒	Animals and other organisms
☒	☐	Human research participants
☒	☐	Clinical data
☒	☐	Dual use research of concern

Methods

n/a	Involved in the study	
☒	☐	ChIP-seq
☒	☐	Flow cytometry
☒	☐	MRI-based neuroimaging

Animals and other organisms

Policy information about studies involving animals; ARRIVE guidelines recommended for reporting animal research

Laboratory animals	The following information comes from Winnubst et. al. 2019, which describes how the mouse brain images were acquired: Wild-type C57BL/6 animals were obtained from Charles River Laboratories. Rorb-IRES2-Cre (IMSR_JAX: 023526;Harris et al., 2014)and Sim1-Cre (IMSR_JAX: 006451;Balthasar et al., 2005) transgenic animals were obtained from The Jackson Laboratory. Adult females (>8 weeks) were used for all experiments and were group housed with sex-matched littermates.
Wild animals	No wild animals used
Field-collected samples	No field collected samples
Ethics oversight	All experimental protocols were conducted according to the National Institutes of Health guidelines for animal research and were approved by the Institutional Animal Care and Use Committee at Howard Hughes Medical Institute, Janelia Research Campus (Protocol #14–115).

Note that full information on the approval of the study protocol must also be provided in the manuscript.
